# Association between physical activity and depression in university students: a systematic review and meta-analysis with public health implications

**DOI:** 10.3389/fpsyg.2026.1850043

**Published:** 2026-07-20

**Authors:** Yu Wang, Haiyu Mao, Xueyun Zhang, Dekun Li, Zhenkai Liu, Wei Shang, Guoqiang Song

**Affiliations:** 1 Education Department, Dongshin University, Naju, Republic of Korea; 2 Graduate School of Education, Yonsei University, Seodaemun-gu, Republic of Korea; 3 Department of Optometry, Dongshin University, Naju, Republic of Korea; 4 Department of Orthopaedics, Shiyan People’s Hospital Affiliated to Hubei University of Medicine, Shiyan, China; 5 Physical Education School, Hanjiang Normal University, Shiyan, China

**Keywords:** depression, meta-analysis, physical activity, sedentary behavior, systematic review, university students

## Abstract

**Objective:**

To systematically review and meta-analyze the association between physical activity and depression among university students, quantify the linear correlation between physical activity level and depressive symptoms, and examine the binary association of insufficient physical activity or sedentary behavior with depression risk, thereby providing evidence-based support for mental health promotion and public health interventions in higher education settings.

**Methods:**

Following PRISMA guidelines, we systematically searched PubMed, Web of Science, Embase, Scopus, and PsycINFO from inception to March 31, 2026, for observational studies examining the relationship between physical activity and depression in university students. Two reviewers independently performed study selection, data extraction, and quality assessment. For studies reporting correlation coefficients, separate meta-analyses were conducted for Pearson and Spearman correlations using Fisher’s z-transformation under a random-effects model. For studies reporting binary outcomes, adjusted odds ratios (ORs) with 95% confidence intervals were extracted and pooled separately for physical activity and sedentary behavior groups.

**Results:**

A total of 36 studies were included: 30 in the linear correlation meta-analysis and 6 in the binary association meta-analysis. The linear correlation analysis comprised 31 effect sizes with a total sample of 30,307 individuals. The pooled correlation estimates showed an inverse direction but substantial to extreme heterogeneity (Pearson: r = −0.19, 95% CI: −0.28 to −0.11, I^2^ = 94.4%; Spearman: r = −0.44, 95% CI: −0.73 to −0.01, I^2^ = 99.2%), indicating that these pooled estimates should be interpreted as average directional trends rather than precise or universally applicable effect sizes. After trim-and-fill correction, the Pearson correlation remained statistically significant (r = −0.114, 95% CI: −0.209 to −0.016). The binary association analysis showed that the pooled OR for the physical activity group (4 studies) was 0.53 (95% CI: 0.27 to 1.07), suggesting a protective trend of higher physical activity against depression that did not reach statistical significance; the pooled OR for the sedentary behavior group (2 studies) was 1.60 (95% CI: 1.26 to 2.04), indicating a significant association between higher sedentary behavior and increased depression risk. Subgroup analyses revealed a stronger negative association between physical activity and depression during and after the COVID-19 period.

**Conclusion:**

Physical activity promotion and sedentary behavior reduction may be practical components of campus mental health strategies, including brief activity breaks during long lectures, low-threshold exercise opportunities, and active commuting support. However, because most included studies were cross-sectional, geographically skewed toward Chinese samples, and highly heterogeneous, these implications should be interpreted cautiously as public health directions rather than causal or universally generalizable recommendations.

## 1 Introduction

Depression is one of the most common and burdensome mental disorders worldwide. It significantly impairs emotional, cognitive, and social functioning and is closely linked to self-harm, increased suicide risk, academic impairment, and long-term decline in quality of life (GBD 2019 Diseases and Injuries Collaborators, 1990; Malhi and Mann, 2018). The university stage represents a critical transition from late adolescence to early adulthood, during which individuals face multiple challenges including academic competition, interpersonal adaptation, career planning, and financial pressure, making them more vulnerable to emotional disorders. Previous international surveys have shown that the burden of common mental disorders among university students is considerable, and depressive symptoms have become an important topic in college mental health and student health management (Auerbach et al., 2017; Ibrahim et al., 2013). Among Chinese university students, the prevalence of depression also remains high, indicating persistent pressure on mental health prevention and control in higher education (Gao et al., 2020).

Physical activity, as a low-cost, scalable health behavior with both physical and mental benefits, has been recognized by the World Health Organization as a key strategy for promoting overall health and preventing non-communicable diseases (WHO, 2020). In addition to improving cardiorespiratory fitness, weight control, and metabolic health, accumulating evidence suggests that physical activity may protect against depression through mechanisms such as regulating neurotransmitters, reducing inflammation, improving sleep quality, and enhancing self-efficacy and social connectedness (Schuch et al., 2018; Kandola et al., 2019). These pathways can also be understood within a behavioral activation framework: depressive symptoms are often accompanied by avoidance, social withdrawal, reduced daily structure, and fewer rewarding experiences, whereas regular physical activity may increase routine, mastery, social interaction, and exposure to positive reinforcement. This framework is particularly relevant to university students, whose mental health is strongly shaped by academic schedules, peer networks, sedentary study patterns, and campus-based opportunities for health behavior change. In the general adult population, systematic reviews and meta-analyses of prospective studies have indicated that higher levels of physical activity are associated with a lower risk of incident depression, with some dose–response relationship (Pearce et al., 2022). Recent cross-sectional evidence from U. S. adults has also shown that higher vigorous physical activity and lower sedentary behavior were associated with fewer depressive symptoms, further supporting the relevance of movement behaviors to mental health across populations (Huang and Huang, 2023). Therefore, from a public health perspective, promoting physical activity may be a feasible pathway to reduce the burden of depression.

However, directly extrapolating evidence from general adults to university students has limitations. First, university students have a distinct lifestyle characterized by prolonged sedentary study time, increased screen exposure, irregular sleep patterns, and insufficient exercise, all of which may intertwine with depression. Light physical activity may benefit mood by interrupting inactivity, increasing energy expenditure, improving affective regulation, and providing low-threshold opportunities for social or outdoor engagement. In contrast, reducing sedentary behavior may operate through a partly different pathway by decreasing prolonged sitting, screen exposure, social isolation, and sleep disruption, even when it does not necessarily increase moderate-to-vigorous physical activity (WHO, 2020). Second, physical activity patterns during university are highly heterogeneous; different studies vary considerably in activity type, frequency, intensity, measurement tools, and depression assessment methods, leading to inconsistent conclusions. Third, most primary studies on the relationship between physical activity and depression in university students are cross-sectional, making them susceptible to reverse causation and residual confounding. Depressive symptoms themselves may lead to reduced activity, thereby affecting the interpretation of the association strength (Pearce et al., 2022). Furthermore, differences in gender, cultural background, academic major, and post-pandemic campus environments may further exacerbate inconsistencies across studies.

Although a recent systematic review and meta-analysis has examined the relationship between physical activity and depression in college students, important methodological issues remain unresolved, including the handling of different correlation metrics, the instability introduced by extreme heterogeneity, and the limited integration of sedentary behavior and binary risk estimates (Huang et al., 2025). The present study extends the available evidence by applying a dual-track analytical framework, separating Pearson and Spearman correlation analyses, and additionally synthesizing binary associations for physical activity and sedentary behavior.

Based on this, the present study aimed to systematically review existing observational evidence using a dual-track analytical framework: on one hand, to quantitatively synthesize the linear correlation between physical activity level and depressive symptoms; on the other hand, to examine the binary association of insufficient physical activity with depression risk using defined thresholds. Through this multi-dimensional meta-analysis, we hope not only to provide objective evidence for identifying potential physical–mental dose–response relationships but also to offer practical scientific insights for campus health interventions, graded depression prevention, and public health response strategies.

## 2 Methods

### 2.1 Study design

This systematic review and meta-analysis aimed to comprehensively evaluate the association between physical activity and depression among university students and to further examine the binary associations of insufficient physical activity or sedentary behavior with depression risk. The study design, literature screening, data extraction, and result reporting followed the Preferred Reporting Items for Systematic Reviews and Meta-Analyses (PRISMA) guidelines.

### 2.2 Search strategy

We systematically searched PubMed, Web of Science, Embase, Scopus, and PsycINFO from inception to March 31, 2026, for observational studies examining the relationship between physical activity and depression in university students. To maximize sensitivity, we supplemented the search with reference tracing of included articles. The search strategy combined subject headings and free text terms around three core concepts: (1) study population: college students, university students, undergraduates, graduates; (2) exposure: physical activity, exercise, sport, sedentary behavior, sedentary time; (3) outcome: depression, depressive symptoms.

An example PubMed search string was: (“college student” OR “*university student*” OR undergraduate* OR graduate*) AND (“physical activity” OR exercise OR sport OR “sedentary behavior” OR “sedentary time”) AND (depression OR “depressive symptoms”).

### 2.3 Inclusion and exclusion criteria

#### 2.3.1 Inclusion criteria

Studies were included if they met all the following criteria: (1) Population: regular university students or postgraduates, including undergraduates, college students, medical students, freshmen, and other enrolled university students; (2) Study type: observational studies, including cross-sectional, cohort, and longitudinal studies, as well as baseline cross-sectional data from randomized controlled trials; (3) Exposure: reported physical activity, exercise behavior, or sedentary behavior; physical activity could be measured by self-report questionnaires or objective devices; (4) Outcome: depression or depressive symptoms assessed using standardized scales, such as BDI/BDI-II, PHQ-9, SDS, CES-D, or the depression subscale of DASS; (5) Effect size extractable: studies reported a correlation coefficient (Pearson or Spearman) between physical activity and depression, or reported a binary association effect size (e.g., odds ratio with 95% confidence interval), or provided raw data allowing calculation; (6) Document type: full-text articles published in English or Chinese.

#### 2.3.2 Exclusion criteria

Studies were excluded if they met any of the following criteria: (1) Study population not university students, or data for university students could not be extracted from a mixed sample; (2) No reported association between physical activity/sedentary behavior and depression, and raw data for calculation could not be obtained; (3) Review, conference abstract, dissertation, commentary, case report, protocol paper, or duplicate publication; (4) Use of non-standard depression assessment methods, or unclear definitions of exposure or outcome; (5) For duplicate publications, only the most complete or largest sample version was retained; (6) Full text not obtainable.

### 2.4 Study selection

All retrieved records were imported into reference management software for deduplication. Two reviewers independently performed title and abstract screening followed by full-text review according to the pre-specified inclusion and exclusion criteria. Disagreements between the two reviewers were resolved through discussion or by consulting a third reviewer. The screening process is presented as a PRISMA flow diagram.

For studies reporting data at multiple time points, if each time point could be considered a relatively independent observation, they were recorded as separate effect sizes; the independence of samples and time structure was assessed to avoid double-counting.

### 2.5 Data extraction

Two reviewers independently extracted data from included studies, including: first author, publication year, country/region, study design, sample size, participant characteristics, depression measure, physical activity measure, statistical analysis methods, survey period, and effect size information.

For the linear correlation meta-analysis, we extracted correlation coefficients (r) and corresponding sample sizes, either Pearson or Spearman. Reported correlation coefficients were converted to Fisher’s z values and standard errors for pooling, and then back-transformed to r for presentation to enhance interpretability.

For the binary association meta-analysis, we extracted multivariable-adjusted odds ratios (adjusted OR) with 95% confidence intervals. If only regression coefficients, standard errors, or raw frequencies were reported, we converted them to OR and corresponding standard error where possible. For studies with different exposure directions (e.g., “high physical activity vs. low” vs. “low physical activity vs. high”), we harmonized the direction so that OR > 1 indicated increased depression risk and OR < 1 indicated reduced risk.

Because included studies varied in participant descriptions, physical activity measures, depression measures, and definitions of the COVID-19 period, we standardized coding for key variables: (1) Study population: categorized as general university students, medical students, freshmen, postgraduates/medical postgraduates, and high-risk samples; (2) Depression measure: categorized as BDI/BDI-II, PHQ-9, SDS, CES-D, DASS-21/42 depression subscale, etc.; (3) Physical activity measure: categorized as PARS-3, IPAQ/IPAQ-SF, GPAQ, device-based measures (e.g., accelerometer, step count), and single-item exercise frequency; (4) Survey period: categorized as pre-COVID-19, during COVID-19, post-COVID-19.

Disagreements in data extraction were resolved by checking the original articles and discussion.

### 2.6 Quality assessment

Methodological quality of included studies was independently assessed by two reviewers using a checklist based on the AHRQ framework for observational studies, adapted to the available items. Ten methodological domains were evaluated: clear objective, defined population, acceptable participation rate, uniform recruitment, justified sample size, valid exposure measurement, valid outcome measurement, blinding, confounding control, and appropriate statistical analysis. Each item was rated as “yes,” “no,” “partial,” or “unclear.” Each domain was rated as “yes,” “no,” “partial,” or “unclear.” In line with domain-based quality assessment principles, we did not use an aggregate numerical score as the primary basis for judging study quality, because limitations in key domains such as confounding control or exposure measurement cannot be fully offset by adequate reporting in other domains. Therefore, study quality was interpreted according to domain-specific ratings, and recurring methodological limitations were summarized across studies. Disagreements were resolved by discussion.

### 2.7 Statistical methods

#### 2.7.1 Linear correlation meta-analysis

For studies reporting correlation coefficients, we separated effect sizes into Pearson correlations and Spearman rank correlations and conducted independent meta-analyses. For Pearson correlations, we used Fisher’s z-transformation for pooling; for Spearman correlations, we also used Fisher’s z-transformation as an approximation (noting that this is an approximation because the standard error estimation for Spearman r has theoretical limitations) and back-transformed to r for reporting. Both analyses used a random-effects model (REML) with Hartung–Knapp correction for 95% confidence intervals. Because the two types of correlation coefficients reflect different statistical relationships, we did not pool them into a single overall effect size but reported them separately and interpreted the consistency of direction in the discussion.

Based on the statistical methods reported in primary studies, correlation studies were divided into Pearson and Spearman groups for separate analyses, and between-group differences were tested. Heterogeneity was assessed using Cochran’s Q test and the I^2^ statistic; higher I^2^ values indicated greater heterogeneity. To explore sources of heterogeneity, we conducted subgroup analyses based on pre-coded study characteristics, including: depression assessment tool, physical activity measurement tool, and survey period relative to COVID-19. For Pearson correlation studies, we further performed meta-regression with the log-transformed sample size (log_n) as a continuous moderator to examine whether sample size explained part of the between-study heterogeneity. Publication bias was assessed only for analyses including at least 10 studies. Therefore, funnel plot inspection and trim-and-fill analysis were performed for the Pearson correlation analysis, whereas publication bias was not formally assessed for the Spearman correlation analysis because fewer than 10 studies were available. To assess the robustness of the correlation meta-analysis, we conducted sensitivity analyses and leave-one-out analyses separately for Pearson and Spearman correlations. In the leave-one-out analysis, each study was sequentially omitted and the pooled random-effects estimate was recalculated. Additional sensitivity analyses were performed by restricting the dataset to studies with directly comparable effect sizes, excluding very small studies (n < 100), and excluding studies with extreme observed correlations (|r| ≥ 0.80).

#### 2.7.2 Binary association meta-analysis

For studies reporting binary outcomes, OR was used as the effect size. ORs were log-transformed and pooled with their standard errors. Given substantial differences across studies in exposure definitions, sample populations, and outcome thresholds, we used a random-effects model (REML) with Hartung–Knapp correction for 95% confidence intervals. Based on exposure type, binary studies were stratified into: (1) PA group: reflecting physical activity level, meeting MVPA threshold, or exercise frequency; (2) SB group: reflecting sedentary time or sedentary behavior level. Heterogeneity was assessed using Q test, I^2^, and τ^2^. Because the number of studies in each binary subgroup was small (PA group, k = 4; SB group, k = 2), publication bias was not assessed and funnel plots were not generated for the binary analyses. All statistical analyses were performed using R software. Two-sided *p* < 0.05 was considered statistically significant.

## 3 Results

### 3.1 Study selection

**Figure 1:**
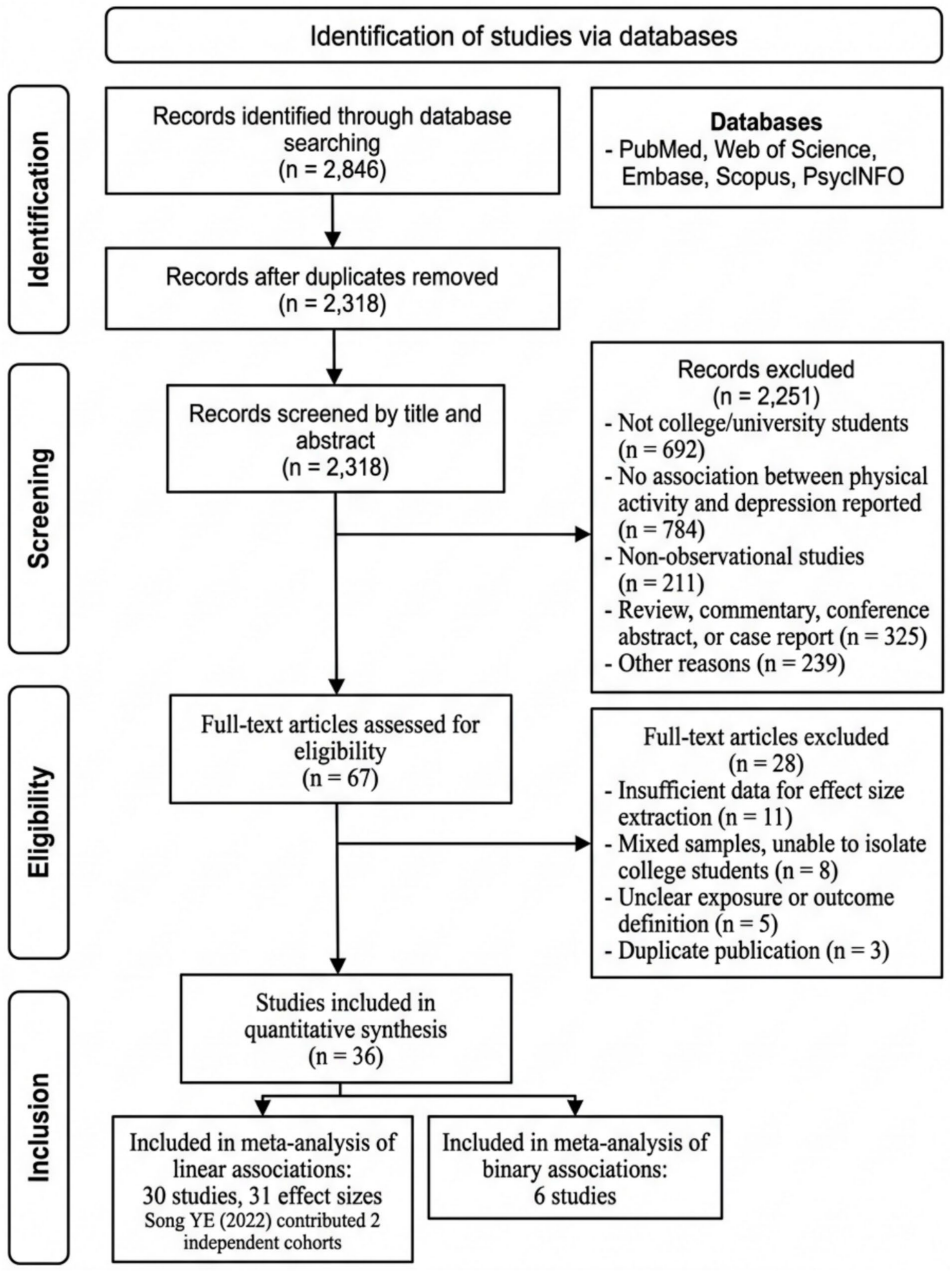
PRISMA 2020 flow diagram of study selection.

A systematic search of PubMed, Web of Science, Embase, Scopus, and PsycINFO up to March 31, 2026, identified 2,846 records. After deduplication, 2,318 records remained; 2,251 were excluded based on title and abstract screening, leaving 67 full-text articles for assessment. Of these, 28 were excluded due to incomplete data, ineligible study population, duplicate publication, or other reasons. Finally, 36 independent studies were included in the quantitative analysis: 30 in the linear correlation meta-analysis (including two independent cohorts reported by Song YE 2022, giving 31 effect sizes) and 6 in the binary association meta-analysis. The study selection process is shown in Figure 1 (PRISMA flow diagram).

### 3.2 Basic characteristics of included studies

Characteristics of included studies are presented in Tables 1, 2. In the linear correlation meta-analysis, 30 studies were included with a total sample size of 30,307 individuals (range 40 to 5,341). Most studies were cross-sectional, with one longitudinal study and one baseline of an RCT. Song YE (2022) reported two independent cohorts (pre-COVID and during COVID), contributing 31 effect sizes in the linear correlation meta-analysis. Study populations included general university students, undergraduates, postgraduates, as well as medical students, freshmen, female students, and samples with high depressive symptom risk. Depression assessment tools included BDI/BDI-II, PHQ-9, SDS, CES-D/CESD-10, DASS-21/42 depression subscale, TDQ, and DEPS. Physical activity measurement tools included PARS-3/PARS, IPAQ/IPAQ-SF, GPAQ, PAQ, Godin-Shephard LTPA, single-item exercise frequency, self-reported exercise, and accelerometers. By effect size type, there were 24 Pearson correlations and 7 Spearman correlations. The binary association meta-analysis included 6 studies with sample sizes ranging from 150 to 8,059; most were cross-sectional, and effect sizes were primarily derived from logistic regression models. Overall, included studies showed heterogeneity in sample characteristics, measurement tools, exposure definitions, outcome thresholds, and survey periods.

**Table 1:** Characteristics of studies included in the meta-analysis of linear associations between physical activity and depression.

Study	Country	Design	n	Mean age	Female (%)	Academic grade/status	Depression measure	PA measure	Corr Type	Period
Zhong et al. (2025)	China	Cross-sectional	3,326	19.38 (NR)	50.9	College students	BDI-II	PARS-3	Pearson	Post-COVID
Cecchini et al. (2019)	Spain	Cross-sectional	353	20.12 (NR)	50.1	College students	Kandel Scale	IPAQ	Pearson	Pre-COVID
Coughenour et al. (2021)	USA	Cross-sectional	194	25.11 (NR)	72.7	Undergraduate and graduate students	PHQ-9	Self-report	Pearson	During COVID
Fu et al. (2023)	China	Cross-sectional	478	NR	53.8	Freshmen	SDS	PARS-3	Spearman	Post-COVID
Gerber et al. (2014)	Switzerland	Cross-sectional	451	22.7 (2.3)	62	Medical and EHS students	Depression Scale	OIMQ	Pearson	Pre-COVID
Han et al. (2023)	China	Cross-sectional	251	20.66 (1.23)	42.2	College students	SDS	IPAQ-SF	Spearman	During COVID
Herbert et al. (2020)	Germany	RCT baseline	141	23.05 (3.54)	83	College students	BDI-II	GPAQ	Pearson	Pre-COVID
Huang C. et al. (2024)	China	Cross-sectional	326	NR	49.1	Undergraduate and graduate students	DASS-21	PARS-3	Pearson	Post-COVID
Kim et al. (2021)	Korea	Cross-sectional	525	NR	50.7	Undergraduates	BDI-II	IPAQ-SF	Pearson	During COVID
Li et al. (2023)	China	Cross-sectional	2,606	19.31 (1.43)	33.2	College students	BDI-II	IPAQ-SF	Pearson	During COVID
Li et al. (2024)	China	Cross-sectional	40	18.51 (0.42)	100	Female freshmen	BDI-II	ActiGraph GT3X	Pearson	Post-COVID
Lin et al. (2022)	Taiwan	Cross-sectional	605	NR	47.1	Undergraduates	TDQ	Fox Scale	Pearson	During COVID
Liu et al. (2023)	China	Cross-sectional	1,292	19.4 (1.24)	49.7	Undergraduates	CES-D-10	PAQ	Pearson	During COVID
Ofili et al. (2024)	Nigeria	Cross-sectional	383	23.08 (3.71)	57.2	Undergraduates	PHQ-9	IPAQ-SF	Spearman	During COVID
Qin et al. (2024)	China	Cross-sectional	5,341	NR	67.9	Undergraduates	DASS-C	PARS-3	Pearson	During COVID
Shimamoto et al. (2021)	Japan	Cross-sectional	85	18.9 (1.4)	38.8	Liberal arts students	PHQ-9	Accelerometer (steps)	Pearson	During COVID
Song and Lim (2022)	South Korea	Cross-sectional	467	NR	100	Female college students	BDI	IPAQ-short	Pearson	Pre-/During COVID
de Souza et al. (2021)	Brazil	Cross-sectional	367	NR	68.1	College students	BDI	IPAQ (MVPA)	Spearman	During COVID
Tang et al. (2022)	China	Cross-sectional	479	19.94 (1.25)	38.8	College students	SDS	PARS-3	Pearson	Post-/During COVID
Vally and Helmy (2021)	Egypt and UAE	Cross-sectional	1,322	19.5 (1.54)	75.4	College students	DASS-21 depression subscale	Single-item exercise frequency	Pearson	During COVID
Wang et al. (2024)	China	Cross-sectional	766	19.1 (1.37)	59	College freshmen	SDS	PARS-3 (modified)	Pearson	During COVID
Wei et al. (2024)	China	Cross-sectional	579	NR	77.2	College students	SDS	PARS-3	Pearson	Post-COVID
Yang et al. (2022)	China	Cross-sectional	586	20.26 (1.78)	43.1	College students	PHQ-9	PARS-3	Spearman	During COVID
Yano and Oishi (2018)	Japan	Cross-sectional	275	19.4 (1.1)	50.9	College students	SDS	Single-item exercise frequency	Pearson	Pre-COVID
Ye et al. (2024)	China	Longitudinal	305	18.54 (NR)	0	Male college students	PHQ-9	Godin-Shephard LTPA	Pearson	Post-COVID
Yu et al. (2025)	China	Cross-sectional	2,537	22.23 (5.03)	54.7	College students	PHQ-9	PARS-3	Pearson	Post-COVID
Yue et al. (2022)	China	Cross-sectional	2,217	NR	68.3	Medical postgraduates	DASS-21 depression subscale	PARS-3	Pearson	During COVID
Zhu et al. (2023)	China	Cross-sectional	442	NR	NR	College students with depressive symptoms	BDI-II	IPAQ-SF	Spearman	During COVID
Dou et al. (2025)	China	Cross-sectional	1,205	19.84 (1.31)	57.8	College students	CESD-10	IPAQ-SF	Spearman	Post-COVID
Ma et al. (2024)	China	Cross-sectional	2,363	NR	44.7	Full-time university students	DEPS	PARS	Pearson	During COVID

**Table 2:** Characteristics of studies included in the meta-analysis of binary associations between physical activity and depression.

Study	Country	Design	n	Mean age	Female (%)	Academic grade/status	Depression definition	PA exposure	Model	Period
Wang X. et al. (2025)	China	Cross-sectional	150	18.96 (0.77)	54	College students	SDS 53–62 vs. < 53	MVPA: yes vs. no	Logistic regression	Post-COVID
Gosadi (2024)	Saudi Arabia	Cross-sectional	506	22 (2.1)	46.8	University students	DASS-21 abnormal vs. normal	Exercise ≥1 time/week vs. none	Logistic regression	Post-COVID
Barbosa et al. (2025)	Brazil	Cross-sectional	8,059	23.9 (6.28)	65.4	Undergraduates	DASS-21 moderate–severe vs. normal/mild	Active vs. inactive PA	Logistic regression	During/Post-COVID
Feng et al. (2014)	China	Cross-sectional	1,106	18.9 (0.9)	42.6	College freshmen	SDS ≥ 53	High vs. low PA	Logistic regression	Pre-COVID
Sotaquirá et al. (2022)	Colombia	Longitudinal	609	17.25 (1)	36.8	First-year undergraduates	BDI-S ≥ 35	Low/moderate/vigorous PA	Logistic regression	Pre-/During-COVID
Wong et al. (2023)	Malaysia	Cross-sectional	388	22.8 (2)	72.4	University students	DASS-21 ≥ mild	Exercise frequency categories	Logistic regression	During COVID

PA, physical activity; PHQ-9, 9-item Patient Health Questionnaire; BDI/BDI-II, Beck Depression Inventory/Beck Depression Inventory-II; SDS, Self-Rating Depression Scale; CES-D, Center for Epidemiologic Studies Depression Scale; DASS-21, Depression Anxiety Stress Scale-21; IPAQ, International Physical Activity Questionnaire; IPAQ-SF, International Physical Activity Questionnaire-Short Form.

### 3.3 Quality assessment

**Figure 2:**
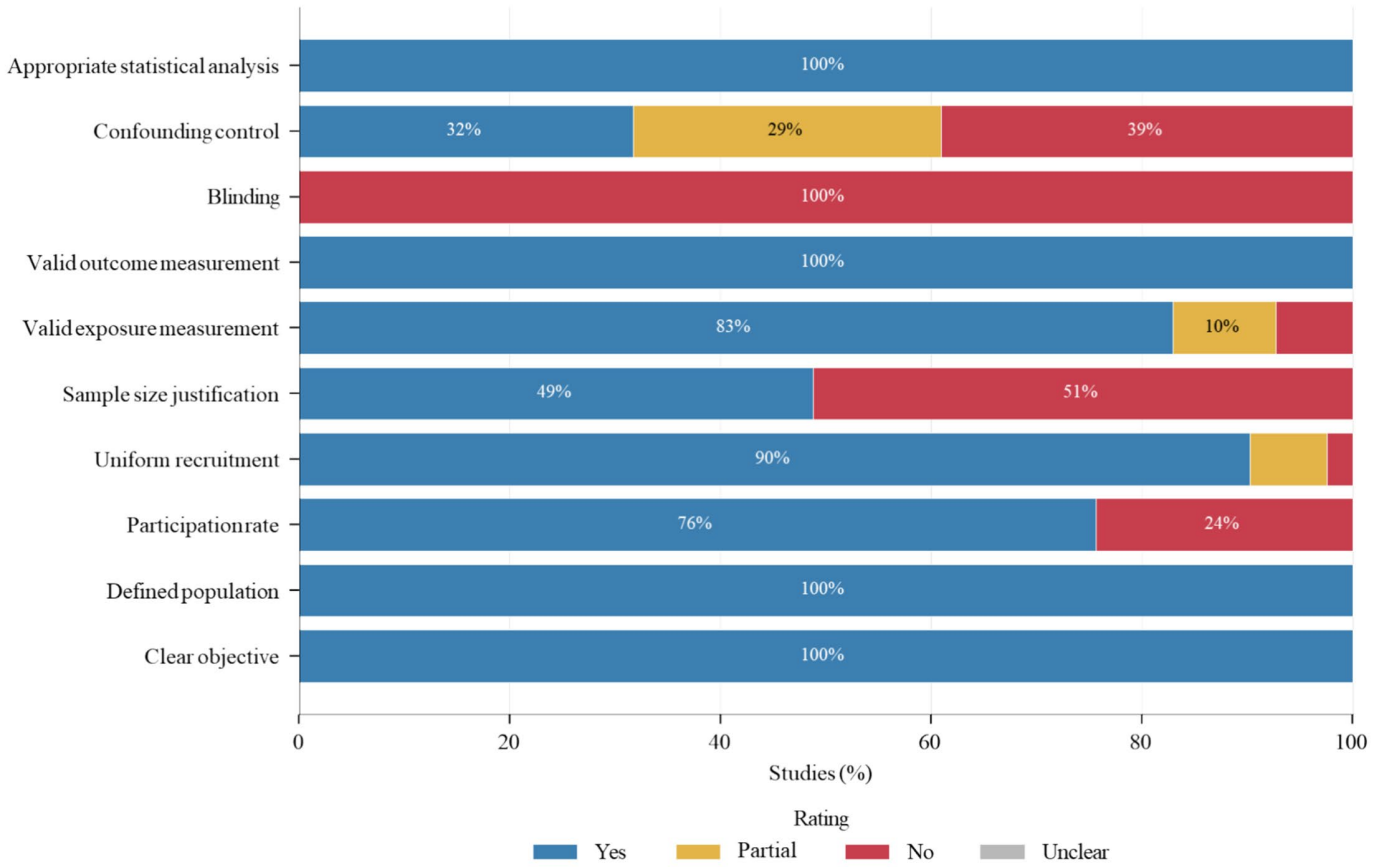
Domain-specific methodological quality assessment using the adapted AHRQ checklist. Bars show the percentage of study-level assessments rated as Yes, Partial, No, or Unclear for each methodological domain. Obj, clear objective; Pop, defined population; PR, participation rate; Rec, uniform recruitment; SSJ, sample size justification; Exp, exposure measurement validity; Out, outcome measurement validity; Blind, blinding; Conf, confounding control; Stat, appropriate statistical analysis.

Study-level methodological quality ratings are shown in Table 3, and the domain-level summary is presented in Figure 2. At the domain level, all study-level assessments clearly reported objectives, defined populations, outcome measurement, and appropriate statistical analysis. However, recurring limitations were observed in blinding, sample size justification, participation rate reporting, and confounding control. Specifically, blinding was not reported or not applicable in all assessments, sample size justification was absent in 51.2%, participation rate was insufficient or not reported in 24.4%, and confounding control was fully adequate in only 31.7% of assessments. Therefore, methodological quality was interpreted according to domain-specific limitations rather than aggregate scores alone.

**Table 3:** Domain-specific methodological quality assessment of included studies using the adapted AHRQ checklist.

Study	Obj	Pop	PR	Rec	SSJ	Exp	Out	Blind	Conf	Stat
Zhong et al. (2025)	Yes	Yes	Yes	Yes	No	Yes	Yes	No	Partial	Yes
Cecchini et al. (2019)	Yes	Yes	No	Yes	No	Yes	Yes	No	Partial	Yes
Coughenour et al. (2021)	Yes	Yes	No	Yes	No	Partial	Yes	No	Partial	Yes
Fu et al. (2023)	Yes	Yes	Yes	Yes	Yes	Partial	Yes	No	Partial	Yes
Gerber et al. (2014)	Yes	Yes	No	Yes	Yes	Partial	Yes	No	Partial	Yes
Han et al. (2023)	Yes	Yes	Yes	Yes	Yes	Yes	Yes	No	Yes	Yes
Herbert et al. (2020)	Yes	Yes	No	Yes	No	Yes	Yes	No	Partial	Yes
Huang C. et al. (2024)	Yes	Yes	Yes	Yes	Yes	Yes	Yes	No	Yes	Yes
Kim et al. (2021)	Yes	Yes	Yes	Yes	No	Yes	Yes	No	Partial	Yes
Li et al. (2023)	Yes	Yes	Yes	Yes	Yes	Yes	Yes	No	Partial	Yes
Li et al. (2024)	Yes	Yes	Yes	Yes	Yes	Yes	Yes	No	Partial	Yes
Lin et al. (2022)	Yes	Yes	Yes	Partial	No	Yes	Yes	No	Partial	Yes
Liu et al. (2023)	Yes	Yes	Yes	Yes	No	Yes	Yes	No	No	Yes
Ofili et al. (2024)	Yes	Yes	Yes	Partial	Yes	Yes	Yes	No	No	Yes
Qin et al. (2024)	Yes	Yes	Yes	Yes	Yes	Yes	Yes	No	No	Yes
Shimamoto et al. (2021)	Yes	Yes	Yes	Yes	No	Yes	Yes	No	Partial	Yes
Song and Lim (2022)	Yes	Yes	Yes	Yes	No	Yes	Yes	No	No	Yes
de Souza et al. (2021)	Yes	Yes	No	Yes	Yes	Yes	Yes	No	No	Yes
Tang et al. (2022)	Yes	Yes	Yes	Yes	No	Yes	Yes	No	No	Yes
Vally and Helmy (2021)	Yes	Yes	No	Yes	No	No	Yes	No	No	Yes
Wang et al. (2024)	Yes	Yes	Yes	Yes	Yes	Yes	Yes	No	No	Yes
Wei et al. (2024)	Yes	Yes	Yes	Yes	Yes	Yes	Yes	No	No	Yes
Yang et al. (2022)	Yes	Yes	Yes	Partial	Yes	Yes	Yes	No	No	Yes
Yano and Oishi (2018)	Yes	Yes	No	Yes	No	No	Yes	No	No	Yes
Ye et al. (2024)	Yes	Yes	Yes	Yes	No	Yes	Yes	No	No	Yes
Yu et al. (2025)	Yes	Yes	Yes	Yes	Yes	Yes	Yes	No	No	Yes
Yue et al. (2022)	Yes	Yes	Yes	Yes	No	Yes	Yes	No	No	Yes
Zhu et al. (2023)	Yes	Yes	Yes	Yes	No	Yes	Yes	No	No	Yes
Dou et al. (2025)	Yes	Yes	Yes	Yes	No	Yes	Yes	No	Yes	Yes
Ma et al. (2024)	Yes	Yes	No	Yes	No	Yes	Yes	No	Yes	Yes
Wang X. et al. (2025)	Yes	Yes	Yes	Yes	Yes	Yes	Yes	No	Yes	Yes
Gosadi (2024)	Yes	Yes	No	Yes	Yes	No	Yes	No	Yes	Yes
Barbosa et al. (2025)	Yes	Yes	Yes	Yes	No	Yes	Yes	No	Yes	Yes
Feng et al. (2014)	Yes	Yes	Yes	Yes	Yes	Yes	Yes	No	Yes	Yes
Sotaquirá et al. (2022)	Yes	Yes	Yes	Yes	Yes	Yes	Yes	No	Yes	Yes
Wong et al. (2023)	Yes	Yes	No	Yes	Yes	Yes	Yes	No	Yes	Yes

Methodological quality was assessed using an adapted AHRQ checklist across 10 domains. Each domain was rated as “Yes,” “No,” “Partial,” or “Unclear.” The assessment emphasizes domain-specific methodological limitations rather than an aggregate numerical score. Obj, clear objective; Pop, defined population; PR, participation rate; Rec, uniform recruitment; SSJ, sample size justified; Exp, exposure valid; Out, outcome valid; Blind, blinding; Conf, confounding control; Stat, statistical analysis appropriate; NR, not reported.

Overall, most assessments performed well in reporting objectives, population definitions, outcome measurement, and statistical analysis, but interpretation of the pooled findings should consider recurring domain-specific limitations, especially incomplete confounding control, insufficient sample size justification, lack of blinding, and incomplete participation rate reporting.

### 3.4 Correlation coefficient analysis

#### 3.4.1 Overall analysis results

**Figure 3:**
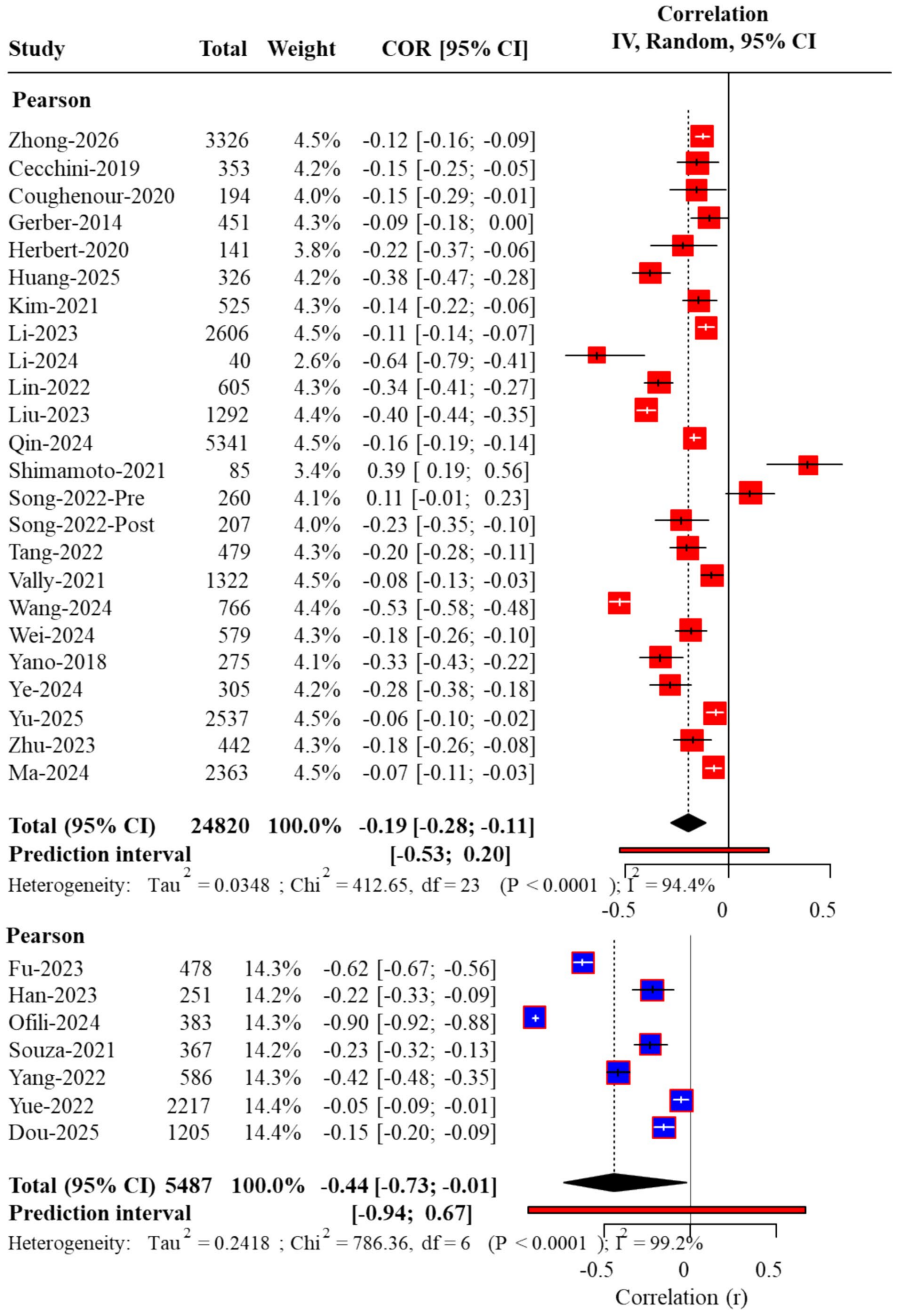
Forest plots of pooled correlations between physical activity and depression with 95% confidence intervals and 95% prediction intervals. Panel **(A)** presents Pearson correlation coefficients; Panel **(B)** presents Spearman correlation coefficients. The diamond represents the pooled random-effects estimate and its 95% confidence interval. The prediction interval indicates the expected range of true effects in future comparable studies.

For 24 Pearson correlation studies, the pooled correlation coefficient was r = −0.19 (95% CI: −0.28 to −0.11), with substantial heterogeneity (I^2^ = 94.4%). The 95% prediction interval was wide and crossed the null value (−0.53 to 0.20), indicating that the true effect in a future comparable study may vary considerably. For 7 Spearman correlation studies, the pooled coefficient was r = −0.44 (95% CI: −0.73 to −0.01), with extreme heterogeneity (^I2^ = 99.2%). The corresponding 95% prediction interval was very wide and also crossed the null value (−0.95 to 0.71), suggesting substantial instability and limited generalizability of the pooled Spearman estimate. Therefore, the pooled correlation estimates should be interpreted as average directional trends rather than precise universal effects. Forest plots with 95% confidence intervals and 95% prediction intervals are shown in Figure 3.

**Figure 4:**
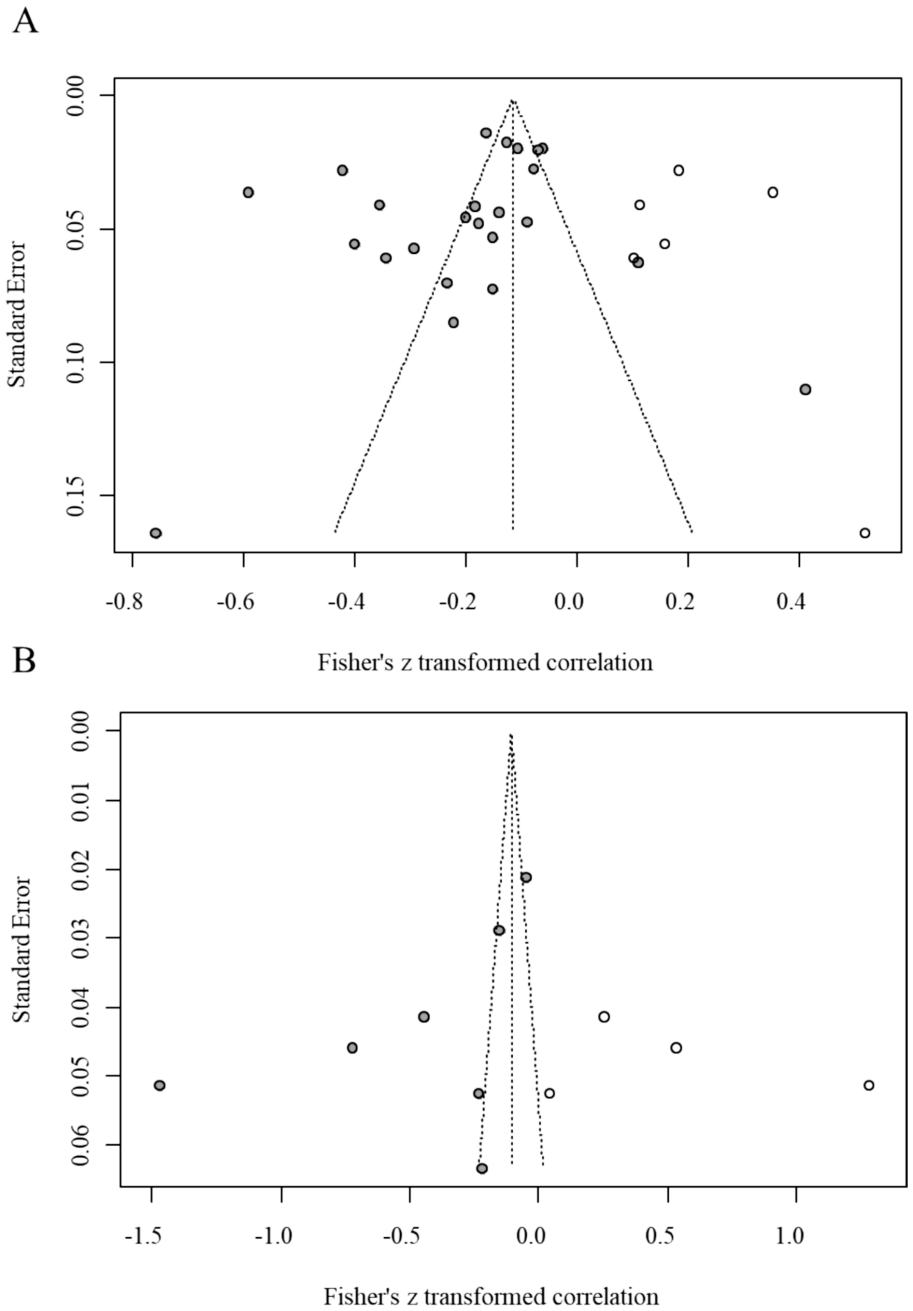
Funnel plot and trim-and-fill analysis for Pearson correlation studies. Panel **(A)** shows Pearson correlations; Panel **(B)** shows Spearman correlations. Publication bias was assessed only for the Pearson correlation analysis because it included more than 10 studies.

Because the Pearson correlation analysis included more than 10 studies, publication bias was assessed using funnel plot inspection and trim-and-fill analysis. The trim-and-fill analysis estimated that 6 missing studies would be needed to achieve funnel plot symmetry; the corrected pooled effect remained statistically significant (r = −0.114, 95% CI: −0.209 to −0.016, *p* = 0.024), indicating a mild negative association with limited influence of small-study effects. Publication bias was not formally assessed for the Spearman correlation analysis because fewer than 10 studies were available. The Pearson funnel plot and trim-and-fill analysis are shown in Figure 4.

#### 3.4.2 Sensitivity and leave-one-out analyses

Sensitivity analyses supported the robustness of the Pearson correlation findings. When the analysis was restricted to studies with directly comparable effect sizes, the pooled Pearson correlation remained negative and statistically significant (r = −0.155, 95% CI: −0.258 to −0.049, *p* = 0.007), although heterogeneity remained high (I^2^ = 93.8%). Excluding very small studies (*n* < 100) yielded a similar result (r = −0.199, 95% CI: −0.263 to −0.134, *p* < 0.001; I^2^ = 94.4%). Excluding extreme observed correlations (|r| ≥ 0.80) did not materially change the estimate (r = −0.193, 95% CI: −0.276 to −0.108, *p* < 0.001; I^2^ = 94.4%). For Spearman correlations, the direction of association remained negative across sensitivity analyses, but the estimates were less stable. Restricting to studies with directly comparable effect sizes produced a negative but non-significant pooled estimate (r = −0.481, 95% CI: −0.789 to 0.020, *p* = 0.056; I^2^ = 99.3%). Excluding very small studies did not change the result because all Spearman studies had n ≥ 100 (r = −0.439, 95% CI: −0.730 to −0.014, *p* = 0.045; I^2^ = 99.2%). After excluding the extreme correlation reported by Ofili-2024, the pooled estimate was attenuated but remained statistically significant (r = −0.295, 95% CI: −0.508 to −0.047, *p* = 0.029), with heterogeneity still high (I^2^ = 97.7%). Sensitivity analyses for pooled Pearson and Spearman correlations between physical activity and depressive symptoms are shown in Table 4.

**Table 4:** Sensitivity analyses for pooled Pearson and Spearman correlations between physical activity and depressive symptoms.

Correlation type	Sensitivity analysis	k	Pooled r (95% CI)	*p* value	I^2^ (%)
Pearson	Comparable effect sizes only	16	−0.155 (−0.258 to −0.049)	0.007	93.8
Pearson	Excluding studies with n < 100	22	−0.199 (−0.263 to −0.134)	<0.001	94.4
Pearson	Excluding studies with |r| > = 0.80	24	−0.193 (−0.276 to −0.108)	<0.001	94.4
Spearman	Comparable effect sizes only	6	−0.481 (−0.789 to 0.020)	0.056	99.3
Spearman	Excluding studies with n < 100	7	−0.439 (−0.730 to −0.014)	0.045	99.2
Spearman	Excluding studies with |r| > = 0.80	6	−0.295 (−0.508 to −0.047)	0.029	97.7

**Figure 5:**
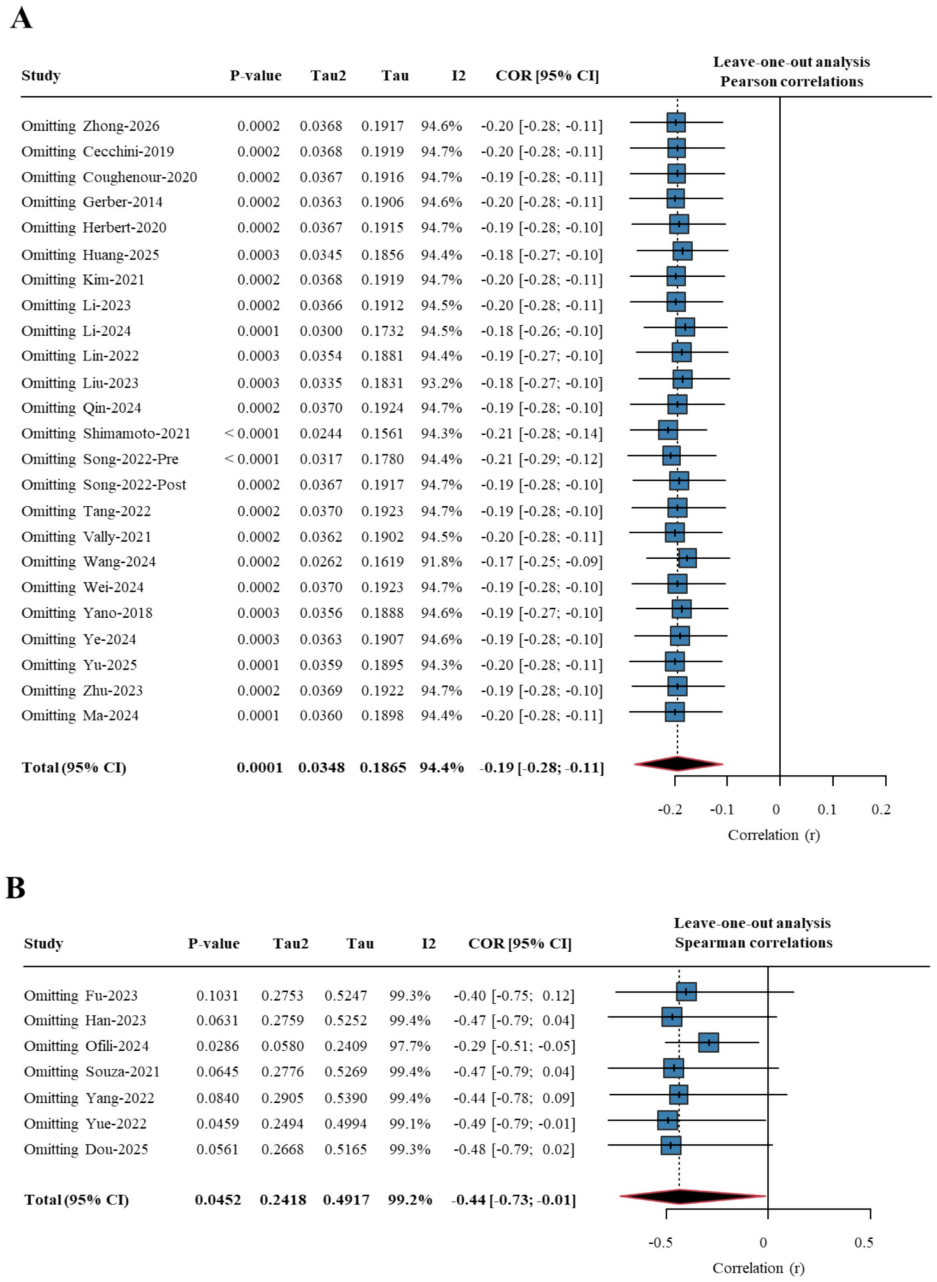
Leave-one-out forest plots for pooled correlations between physical activity and depressive symptoms. Panel **(A)** shows Pearson correlations; Panel **(B)** shows Spearman correlations.

Leave-one-out analyses showed that the pooled Pearson correlation remained consistently negative and statistically significant after omitting each individual study, with pooled r values ranging from −0.211 to −0.175 and I^2^ values ranging from 91.8 to 94.7%. This suggests that the Pearson result was not driven by any single study. For Spearman correlations, all leave-one-out estimates remained negative, with pooled r values ranging from −0.494 to −0.295; however, statistical significance and confidence interval width varied across iterations, and heterogeneity remained very high (I^2^ range: 97.7 to 99.4%). These findings indicate that the Spearman result was directionally consistent but statistically unstable. Leave-one-out forest plots for pooled correlations between physical activity and depressive symptoms are shown in Figure 5.

#### 3.4.3 Subgroup analysis for Pearson correlations

When stratified by depression assessment tool, all subgroups showed negative trends. BDI (r = −0.1704, 95% CI: −0.3305 to −0.0007) and SDS (r = −0.3189, 95% CI: −0.5605 to −0.0273) reached statistical significance. The PHQ-9 subgroup showed a weaker non-significant effect (r = −0.0343, 95% CI: −0.4685 to 0.4133). Single studies using DASS-Total or CES-D showed larger effects but require cautious interpretation. When stratified by physical activity measurement tool, PARS (r = −0.2182, 95% CI: −0.3569 to −0.0702) and Other PA scales (r = −0.2548, 95% CI: −0.4737 to −0.0061) showed significant negative correlations; IPAQ and Device-based/Single-item subgroups showed non-significant effects with wide confidence intervals, suggesting that measurement tools may affect effect stability. When stratified by survey period relative to COVID-19, Post-COVID (r = −0.2617, 95% CI: −0.4642 to −0.0332) and During-COVID (r = −0.2208, 95% CI: −0.3138 to −0.1235) showed significant negative correlations, whereas the Pre-COVID subgroup showed a weaker non-significant correlation (r = −0.0556, 95% CI: −0.3208 to 0.2178). Results of subgroup analyses for Pearson correlations are shown in Table 5.

**Table 5:** Subgroup analysis results for Pearson correlation coefficients.

Subgroup		k	Pooled correlation coefficient r	95% CI	I^2^
Depression assessment tool	BDI	8	−0.1704	−0.3305 ~ −0.0007	80.0%
	Other depression scales	4	−0.1639	−0.3570 ~ 0.0426	92.4%
	PHQ-9	4	−0.0343	−0.4685 ~ 0.4133	91.5%
	DASS-Total	1	−0.3790	−0.4683 ~ −0.2820	—
	CES-D	1	−0.3990	−0.4439 ~ −0.3521	—
	DASS-Dep	2	−0.1246	−0.5733 ~ 0.3816	86.4%
	SDS	4	−0.3189	−0.5605 ~ −0.0273	95.8%
PA measurement tool	PARS	8	−0.2182	−0.3569 ~ −0.0702	96.6%
	IPAQ	6	−0.1168	−0.2307 ~ 0.0003	73.1%
	Other PA scales	4	−0.2548	−0.4737 ~ −0.0061	93.0%
	GPAQ	1	−0.2200	−0.3718 ~ −0.0568	—
	Device-based	2	−0.1653	−1.0000 ~ 1.0000	97.1%
	Single-item	2	−0.2031	−0.9539 ~ 0.8979	93.6%
	Godin-LTPA	1	−0.2840	−0.3841 ~ −0.1773	—
Relationship with COVID-19	Post-COVID	6	−0.2617	−0.4642 ~ −0.0332	91.5%
	Pre-COVID	6	−0.0556	−0.3208 ~ 0.2178	90.4%
	During-COVID	12	−0.2208	−0.3138 ~ −0.1235	96.1%

#### 3.4.4 Subgroup analysis for spearman correlations

Among Spearman correlation studies (k = 7), the random-effects model showed a significant negative overall correlation (r = −0.439, 95% CI: −0.730 to −0.014, *p* = 0.045) but with extremely high heterogeneity (I^2^ = 99.2%). When stratified by depression assessment tool, subgroup differences were significant (Qb = 19.71, *p* = 0.0006). BDI, DASS-Dep, and CES-D subgroups each contained single studies with negative effects; SDS and PHQ-9 subgroups had extremely wide confidence intervals crossing zero, indicating unstable estimates. When stratified by physical activity measurement tool (PARS vs. IPAQ), the subgroup difference was not significant (Qb = 0.09, *p* = 0.7619); both showed negative correlations but with wide confidence intervals. When stratified by COVID-19 period (Post-COVID vs. During-COVID), the subgroup difference was also not significant (Qb = 0.01, *p* = 0.9042); both periods showed consistent direction but unstable estimates. Results of subgroup analyses for Spearman correlations are shown in Table 6.

**Table 6:** Subgroup analysis results for Spearman correlation coefficients.

Subgroup		k	Pooled correlation coefficient r	95% CI	I^2^
Depression assessment tool	SDS	2	−0.4422	−0.9988 ~ 0.9918	97.6%
	PHQ-9	2	−0.7432	−1.0000 ~ 1.0000	99.6%
	BDI	1	−0.2300	−0.3247 ~ −0.1307	—
	DASS-Dep	1	−0.0520	−0.0934 ~ −0.0104	—
	CES-D	1	−0.1500	−0.2047 ~ −0.0943	—
PA measurement tool	PARS	3	−0.3850	−0.8478 ~ 0.4107	99.1%
	IPAQ	4	−0.4770	−0.9107 ~ 0.4570	99.4%
Relationship with COVID-19	Post-COVID	2	−0.4118	−0.9994 ~ 0.9968	99.1%
	During-COVID	5	−0.4495	−0.8309 ~ 0.2193	99.4%

#### 3.4.5 Meta-regression results

**Figure 6:**
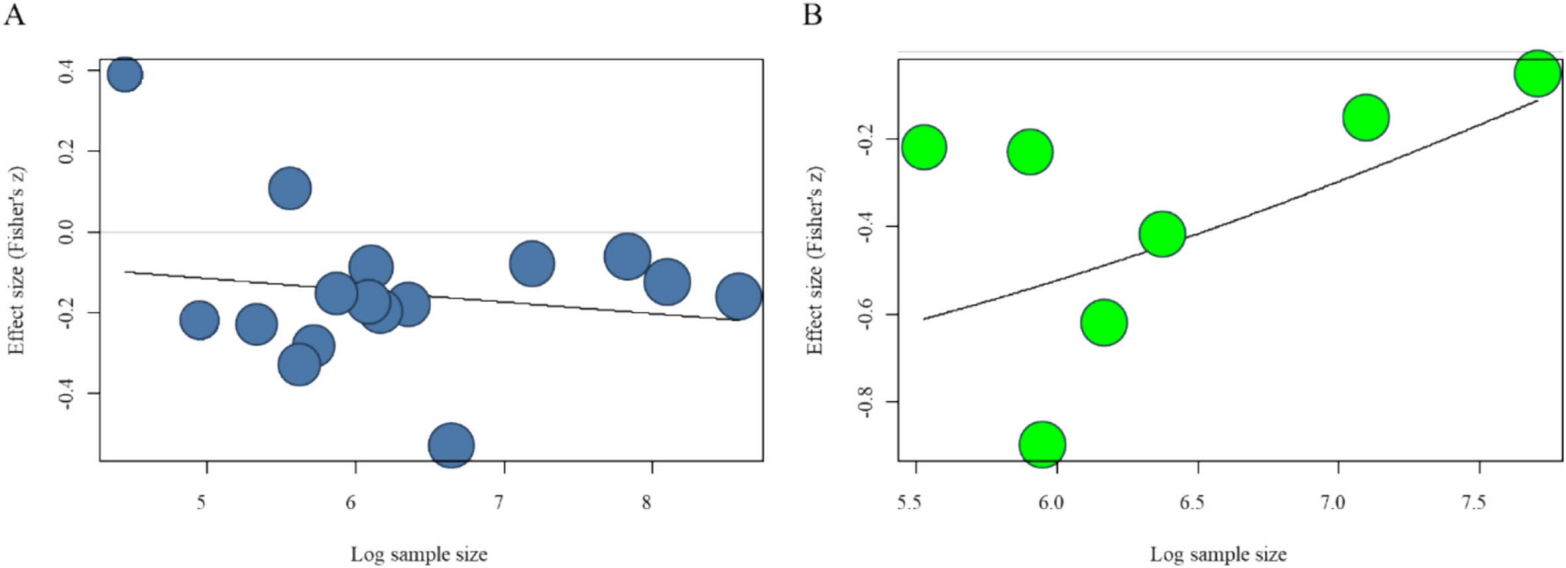
Bubble plot of meta-regression. Panel **(A)** shows Pearson correlations; Panel **(B)** shows Spearman correlations.

In the Pearson correlation subgroup, meta-regression with log-transformed sample size (log_n) as a continuous moderator showed that sample size did not significantly moderate the effect size (*β* = 0.0176, SE = 0.0381, 95% CI: −0.0615 to 0.0966, *p* = 0.649). The overall test of the moderator was not statistically significant (*F* (1, 22) = 0.212, p = 0.649). The model explained 0% of the between-study heterogeneity (R^2^ = 0.00%), and residual heterogeneity remained significant (QE(22) = 378.87, *p* < 0.001; I^2^ = 97.11%), indicating that sample size did not effectively explain the heterogeneity in effect sizes among Pearson correlation studies. The bubble plot of the meta-regression is shown in Figure 6.

### 3.5 Odds ratio analysis

**Figure 7:**
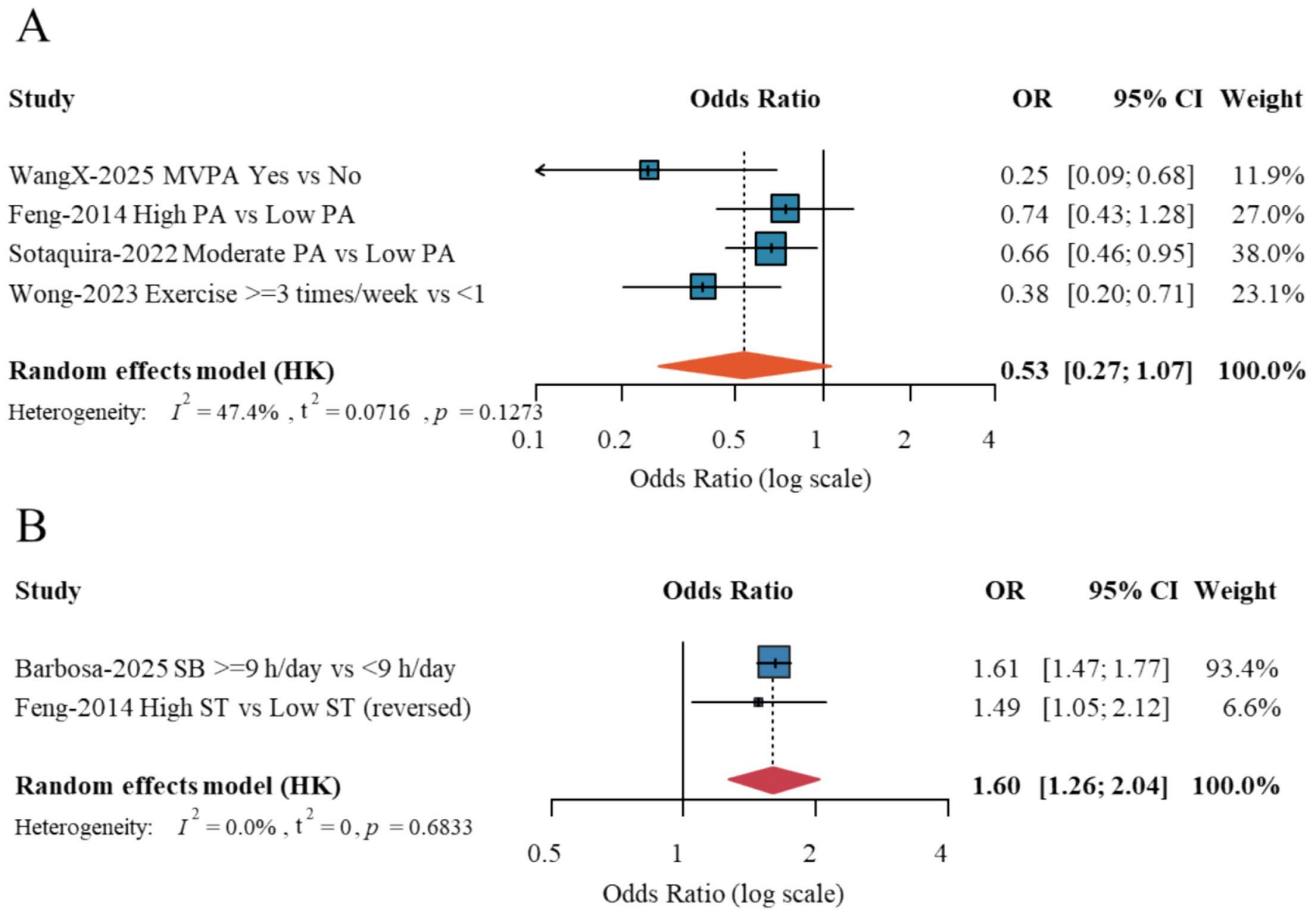
Forest plot of the binary association meta-analysis between physical activity/sedentary behavior and depression. Panel **(A)** shows physical activity; Panel **(B)** shows sedentary behavior.

Two sets of analyses were conducted. For the PA group (4 studies), using a random-effects model (REML) with Hartung–Knapp correction, the pooled OR was 0.53 (95% CI: 0.27 to 1.07, *p* = 0.063), indicating a trend toward reduced depression risk that did not reach statistical significance. Between-study heterogeneity was moderate (I^2^ = 47.4%, τ^2^ = 0.0716), and Cochran’s Q test was not significant (Q = 5.70, df = 3, *p* = 0.127). For the SB group (2 studies), the pooled OR was 1.60 (95% CI: 1.26 to 2.04, *p* = 0.026), indicating a significant association with increased depression risk. Heterogeneity was low (I^2^ = 0%, τ^2^ = 0; Q = 0.17, df = 1, *p* = 0.683). Forest plots for the binary association meta-analysis are shown in Figure 7.

## 4 Discussion

This study systematically synthesized observational evidence on the association between physical activity and depression among university students. The findings suggest a generally inverse association between physical activity and depressive symptoms, but the substantial to extreme heterogeneity indicates that the pooled estimates should be interpreted as average trends rather than stable universal effect sizes. In the binary analysis, higher sedentary behavior was significantly associated with increased depression risk, whereas higher physical activity showed a non-significant protective trend. These findings support physical activity promotion and sedentary time reduction as potentially useful components of campus-based mental health strategies.

The sensitivity and leave-one-out analyses further supported this cautious interpretation. The Pearson correlation findings remained consistently negative and statistically significant, suggesting that the inverse association was not driven by a single influential study. In contrast, the Spearman estimates remained negative in direction but were less stable; therefore, they should be interpreted as supplementary evidence.

Our results are generally consistent with existing evidence in the general population. Previous prospective systematic reviews have shown that higher levels of physical activity are associated with lower risk of incident depression; both early systematic reviews and subsequent large-sample dose–response meta-analyses suggest that even levels below traditional recommended thresholds may confer mental health benefits (Hou et al., 2025; Mammen and Faulkner, 2013; de Oliveira et al., 2026). Moreover, a review on causal inference of physical activity for depression/anxiety concluded that existing evidence generally supports a preventive role of physical activity against depression, although residual confounding and measurement error remain (Wanjau et al., 2023). Thus, the negative association observed in university students is directionally consistent with findings in general adult and prospective cohort studies.

At the same time, our study reveals several characteristics specific to university students. First, the overall heterogeneity was notably high, indicating that university students are not a homogeneous group. Compared with general adults, university students have more phase-specific features such as irregular routines, academic pressure, screen exposure, social adaptation, job anxiety, and campus environment, all of which may modify the strength of the physical activity–depression association (Auerbach et al., 2016). These modifiers may also differ across educational systems and cultural contexts; in highly competitive academic environments, physical activity may function as stress regulation, social interaction, and recovery from prolonged sedentary study, whereas in settings with different campus sports cultures or mental health support systems, the magnitude of the association may differ. Second, our subgroup analysis showed that the negative association between physical activity and depression was stronger during and after the COVID-19 period, whereas the pre-pandemic association was weaker. This finding aligns with the context of increased psychological burden, reduced exercise opportunities, and disrupted daily routines among university students during the pandemic (Kandola et al., 2019; Luo et al., 2021). Existing meta-analyses have shown that the prevalence of depression, anxiety, and stress symptoms among university students increased during the pandemic, and the psychological buffering effect of physical activity may have been more easily observed during this period (Wang et al., 2023).

Our binary analysis also found that sedentary behavior was significantly associated with increased depression risk. This is highly consistent with existing behavioral epidemiological evidence. The World Health Organization’s 2020 guidelines explicitly emphasize that, in addition to encouraging moderate-to-vigorous and moderate-intensity physical activity, sedentary behavior should be minimized (WHO, 2020). Systematic reviews in university students show that this population generally has high levels of sedentary time, with increasing trends over the past decade (Huang et al., 2025). In broader adult populations, systematic reviews on sedentary behavior and depression risk also generally report an unfavorable association, although the magnitude varies by measurement method (Wang D. et al., 2025). Therefore, the overall pattern of “sedentary behavior is harmful, physical activity is beneficial” observed in this study has good external consistency.

Notably, the evidence from linear correlation analyses was stronger than that from binary OR analyses. One possible reason is that dichotomizing physical activity into “high/low” or “meeting/not meeting” thresholds loses substantial continuous information, reducing statistical power. Moreover, definitions of “low activity,” “meeting MVPA,” or “exercise frequency” vary across studies, which can destabilize pooled results. In contrast, correlation analyses retain continuous variation in both exposure and outcome, making it easier to capture dose–response gradients between physical activity and depression in university students. This is consistent with the conclusions of Pearce et al. on dose–response relationships, i.e., even low doses of physical activity may yield meaningful reductions in depression risk (Pearce et al., 2022). However, the correlation evidence should not be interpreted as methodologically uniform. Although Pearson and Spearman coefficients were analyzed separately, the Spearman analysis relied on an approximate standard error using Fisher’s z transformation, included fewer studies, and showed extreme heterogeneity with a wide prediction interval crossing the null value. Therefore, the pooled Spearman estimate should be regarded as supplementary and statistically unstable evidence rather than a precise summary effect.

Furthermore, the direction of our results aligns with interventional studies. Previous systematic reviews and meta-analyses of exercise interventions in clinically depressed patients have shown that exercise can improve depressive symptoms, although effect sizes are influenced by study quality, control conditions, and intervention heterogeneity (Clegg et al., 2026; Stubbs et al., 2018). More relevant to our target population, a recent systematic review and meta-analysis of physical activity interventions in undergraduates also found that such interventions reduce depression, anxiety, and stress levels (Huang K. et al., 2024). Thus, from the chain of evidence from “observational association” to “interventional improvement,” our findings have good biological and practical plausibility.

Our results suggest that mental health prevention and control on campus should not be limited to crisis identification and counseling services but should also incorporate physical activity promotion into campus public health strategies. First, physical activity has the advantages of low barriers, group feasibility, relatively low cost, and few side effects, making it suitable for primary or secondary prevention tools on campus (Stubbs et al., 2018). For university students with mild-to-moderate emotional distress who do not yet meet clinical depression criteria, structured exercise programs, optimized physical education curricula, improved campus walking environments, sports club support, and digital exercise coaching may all be feasible intervention pathways. Second, based on the adverse findings regarding sedentary behavior, campus health promotion should expand from simply “increasing exercise” to a comprehensive model of “increasing activity + reducing sitting.” For example, encouraging activity breaks during classes and study sessions, walking commuting, standing during breaks, active travel between dormitories and classrooms, and reducing prolonged continuous screen exposure may be more helpful for mental health. Third, given that campus environments and student behavioral patterns have changed after the pandemic, universities should place greater emphasis on the role of physical activity in building psychological resilience. Previous research has shown that mental health problems among university students not only affect quality of life but are also associated with academic impairment, dropout risk, and long-term social dysfunction (Auerbach et al., 2016; Zhang et al., 2024). Therefore, integrating physical activity into campus mental health promotion systems may not only reduce the burden of depression but also yield multidimensional benefits in academic performance, sleep, and social adaptation.

This study has several strengths. First, it focuses specifically on university students, avoiding bias from directly extrapolating evidence from general adults to this population. Second, it uses a dual-track analytical framework that simultaneously integrates linear correlation and binary risk analyses, providing a more comprehensive picture of the association between physical activity and depression. Third, subgroup exploration by COVID-19 period, measurement tools, and sample characteristics helps identify potential sources of heterogeneity. Fourth, the domain-specific quality assessment showed generally adequate reporting in several methodological domains, although important limitations remained in confounding control, sample size justification, and blinding.

Several limitations should be acknowledged. First, most included studies were cross-sectional, precluding strong causal inference; reverse causation and residual confounding are inevitable. Second, between-study heterogeneity was high, meaning that the pooled effects should be interpreted as overall trends rather than fixed effects applicable to all campus settings. Third, measurement tools for physical activity and depression varied considerably, and most studies used self-report questionnaires, which may introduce information bias. Fourth, the number of studies in the binary OR analysis was small, and exposure definitions were inconsistent, limiting statistical power and result stability. Fifth, the included studies were geographically dominated by Chinese samples; although there was some international coverage, generalizability to other cultural and educational system contexts requires caution, because academic pressure, campus lifestyle, exercise norms, and mental health support systems may modify the physical activity-depression association. Sixth, publication bias could be assessed only for the Pearson correlation analysis; it was not formally assessed for Spearman or binary analyses because the number of studies was below the recommended threshold for funnel plot asymmetry assessment.

Therefore, future research should prioritize the following: first, more multi-center longitudinal cohort and repeated-measurement studies to clarify the temporal sequence between physical activity and depression; second, use of objective activity monitoring devices alongside standardized depression scales; third, exploration of dose–response relationships, activity type differences, and the combined effects of 24-h behavior composition (activity, sedentary time, sleep) on depression; fourth, more studies on potential moderators such as gender, academic major, grade, economic pressure, and post-pandemic campus environments to generate more targeted intervention recommendations for higher education.

## 5 Conclusion

Physical activity promotion and sedentary behavior reduction should be considered practical components of campus mental health strategies. Rather than relying only on counseling or crisis-oriented services, universities could embed brief activity breaks into long lectures and study sessions, improve access to low-threshold exercise opportunities, support walking or active commuting across campus, and integrate physical activity guidance into student health programs. Given the predominance of cross-sectional evidence, high heterogeneity, and geographical concentration of included studies, these recommendations should be implemented pragmatically and evaluated through longitudinal and intervention studies in diverse educational settings.
